# Brain extracellular matrix retains connectivity in neuronal networks

**DOI:** 10.1038/srep14527

**Published:** 2015-09-29

**Authors:** Arthur Bikbaev, Renato Frischknecht, Martin Heine

**Affiliations:** 1 RG Molecular Physiology, Leibniz Institute for Neurobiology; Brenneckestr. 6, Magdeburg 39118 Germany; 2 RG Brain Extracellular Matrix, Leibniz Institute for Neurobiology; Brenneckestr. 6, Magdeburg 39118 Germany

## Abstract

The formation and maintenance of connectivity are critically important for the processing and storage of information in neuronal networks. The brain extracellular matrix (ECM) appears during postnatal development and surrounds most neurons in the adult mammalian brain. Importantly, the removal of the ECM was shown to improve plasticity and post-traumatic recovery in the CNS, but little is known about the mechanisms. Here, we investigated the role of the ECM in the regulation of the network activity in dissociated hippocampal cultures grown on microelectrode arrays (MEAs). We found that enzymatic removal of the ECM in mature cultures led to transient enhancement of neuronal activity, but prevented disinhibition-induced hyperexcitability that was evident in age-matched control cultures with intact ECM. Furthermore, the ECM degradation followed by disinhibition strongly affected the network interaction so that it strongly resembled the juvenile pattern seen in naïve developing cultures. Taken together, our results demonstrate that the ECM plays an important role in retention of existing connectivity in mature neuronal networks that can be exerted through synaptic confinement of glutamate. On the other hand, removal of the ECM can play a permissive role in modification of connectivity and adaptive exploration of novel network architecture.

Spontaneous activity plays a crucial role in the early development of neuronal networks^1^,^2^,^3^. Network activity appears *in vivo* during the first postnatal week and involves several complementary mechanisms, including GABA mediated depolarization^4^,^5^, intracellular Ca^2+^ transients and non-synaptic activation^6^,^7^. Later in development, the contribution of spontaneous activity to modification of brain connectivity decreases concomitantly with the growing impact of sensory inputs^8^. Nevertheless, early spontaneous activity drives circuitry formation and seems to constitute a fundamental feature of immature neuronal networks both *in vivo* and *in vitro*^2^.

The hyaluronan-based ECM in the brain consists of various chondroitin sulphate proteoglycans, including Aggrecan and Brevican, as well as tenascin-C, tenascin-R and several link proteins, in a complex with hyaluronan acting as a scaffolding backbone^9^,^10^,^11^. Experimental degradation of the ECM was shown to improve post-traumatic regeneration in the brain^12^, restore ocular dominance plasticity^13^, facilitate extinction of fear memory^14^ and enhance cognitive flexibility in reversal learning^15^. Furthermore, knockout mice with reduced ECM expression were found to have juvenile plasticity levels throughout life^16^. Thus, several lines of evidence suggest that ECM is involved in regulation of plasticity and storage of memories in the CNS^17^.

The brain-specific ECM has two appearances: a diffuse ECM that is present throughout the brain and a densely packed ECM referred to as perineuronal nets (PNNs). The adult pattern of ECM/PNNs is an attribute of mature networks and appears both *in vivo*^11^,^18^ and *in vitro*^19^,^20^ during the fourth developmental week as lattice-like structures surrounding predominantly Parvalbumin-positive GABAergic interneurons^18^,^19^,^21^,^22^. Given the tight relationship between neuronal activity and maturation of synaptic connectivity^3^, this led us to assume that manifestation of adult patterns of the network activity and the developmental arrest in cultured neuronal networks^23^,^24^ are linked to activity-dependent^19^ formation of ECM/PNNs. We hypothesized that the ECM in neuronal networks retains the established network connectivity, and thereby preserves optimal conditions for the transfer and processing of information within ensembles of spatially distributed neurons. On the other hand, appearance of the adult ECM ultimately limits the capacity of neuronal network to undergo activity-driven plastic changes.

The density and molecular composition of the ECM varies between different brain areas and the types of neurons it surrounds^10^, therefore the consequences of experimental ECM modification *in vivo* are likely to be region-specific and may substantially vary in degree. Here, we pursued the idea that basic principles of spontaneous formation and maintenance of connectivity are preserved in neuronal cultures with relatively simple and sensory input-free networks^24^. Thus, we tested our hypothesis in high density rat hippocampal cultures grown on microelectrode arrays (MEAs). First, we characterized the developmental profile of spontaneous neuronal activity associated with the formation of network connectivity. We observed that neuronal activity levels and the network interaction properties were stabilized during the fourth week *in vitro*, which coincided with marked increase of the ECM density. Further, in mature neuronal cultures we found that enzymatic degradation of the ECM facilitated the rearrangement of functional network connectivity. Our findings also suggest that ECM integrity in mature neuronal networks contributes to the maintaining the balance between excitation and inhibition, thereby preserving the existing patterns of neuronal network interaction.

## Results

### Stabilization of spontaneous activity levels is accompanied by maturation of the ECM

Spontaneous activity in neuronal cultures is represented by spikes, which reflect single action potentials and population spikes, and bursts, i.e. periods of repetitive neuronal firing followed by periods of almost complete quiescence^25^. First, we monitored the spontaneous neuronal activity of hippocampal cultures over several weeks to characterize its developmental profile in our experimental settings. Consistent with previous reports^26^,^27^,^28^, spikes and bursts were observed by the end of first week *in vitro*, but at this developmental stage the activity was rather scarce and poorly synchronized across the network. Therefore, further analyses were focused on the interval after the 14th day *in vitro* (DIV14), which corresponds to the end of the period dendritic arborisation and spine formation^26^.

**Figure 1:**
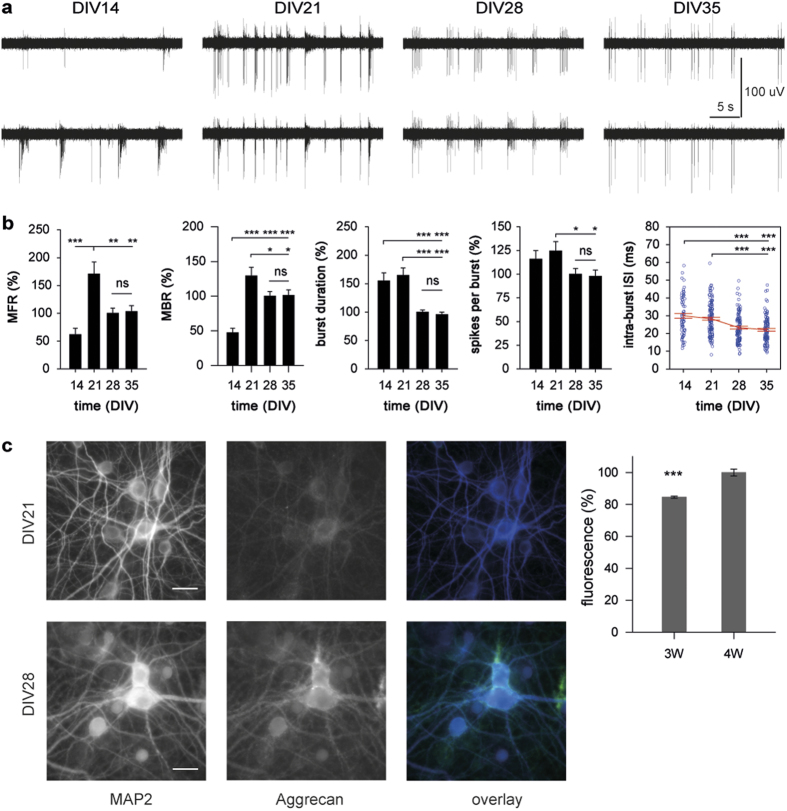
Stabilization of spontaneous activity in neuronal cultures correlates with the increase of ECM density. (**A**) Representative traces of spontaneous neuronal activity derived by two electrodes in the same culture during development. (**B**) The third week *in vitro* was associated with dramatic enhancement, whereas the fourth week of development was associated with overall reduction and the following stabilization of spontaneous neuronal activity. (**C**) The immunoreactivity of Aggrecan was markedly higher in four-week old hippocampal cultures (4W, DIV28-29, n = 30 images from 3 MEAs) as compared to three-week old cultures (3W, DIV21-22, n = 32 images from 3 MEAs). Scale bar 20 μm. *P < 0.05, **P < 0.01, ***P < 0.001; ns: non-significant (Duncan test). Data are shown as mean ± S.E.M.

Spontaneous neuronal activity in all cultures (*n* = 6 MEAs from 5 preparations) was robust within the analyzed period (DIV14-35), but the amount and temporal characteristics of this activity showed much fluctuation. The mean firing rate (MFR) and the mean bursting rate (MBR) varied significantly during development (both P < 0.001 one-way ANOVA), with DIV21 being associated with peak levels of activity (Fig. 1a,b; data were normalized to DIV28 for better comparability across the study). Dramatic enhancement of spontaneous neuronal activity during the third week *in vitro* reflected an increase of network excitability. This could arise through a massive overproduction of glutamatergic synapses and an overall prevalence of excitation over inhibition characteristic for this developmental period^29^. Maturation of excitatory synapses correlates with incorporation of AMPA receptors^30^ that strongly affect temporal characteristics of spike trains^31^, thus the enhanced bursting in three-week old cultures indicated a rather mature state of excitatory connectivity.

Maturation of GABAergic synapses and elimination of excessive glutamatergic connections take place during the fourth week of development^32^,^33^, associated with a marked reduction of both MFR and MBR by DIV28. Inhibitory feedback plays an important role in regulation of burst duration in late development^23^. Significant decreases in mean burst duration (P < 0.001) and the mean number of spikes per burst (P < 0.05) provided further evidence of functional maturation of inhibitory connectivity during the fourth week *in vitro*. The only significant change of these parameters was evident at DIV28: in four-week old cultures, bursts were shorter and comprised on average fewer spikes (P < 0.001 and P < 0.05, respectively; Duncan test) than those in three-week old cultures (Fig. 1b). Hence, the developmental profile of spontaneous neuronal activity faithfully reflected distinct phases of formation and maturation of synaptic connectivity. Importantly, no marked changes of global activity measures were found after DIV28, indicating the settlement of the balance between excitation and inhibition in neuronal cultures upon maturation.

The analysis of spike train properties revealed the lognormal distribution of the mean values per channel for inter-spike (ISI) and inter-burst intervals (IBI) at all developmental stages, whereas the mean intra-burst ISI was characterized by generalized extreme value distribution (Supplementary Fig. S1). The mean intra-burst ISI was significantly affected by the developmental factor (P < 0.001 one-way ANOVA) and markedly decreased during fourth week *in vitro* from 28.3 ± 0.8 ms at DIV21 to 23.3 ± 0.8 ms at DIV28 (P < 0.001 Duncan test) (Fig. 1b). Notably, regardless of developmental stage, topological peculiarities and considerable variability across cultures and preparations, the mean intra-burst ISI remained in the range of 22.1–30.0 ms corresponding to the mean frequency of intra-burst firing in the range of 33.3–45.3 Hz.

To ascertain whether such steady state stabilization of neuronal activity levels during the fourth week *in vitro* was accompanied by changes in ECM density, we analysed immunoreactivity of Aggrecan in hippocampal cultures after three (DIV21–22, n = 3 MEAs) or four (DIV28–29, n = 3 MEAs) weeks *in vitro* (Fig. 1c). We found that mean fluorescence of Aggrecan was markedly higher in four-week old (100.0 ±1.6%, n = 30 images) than three-week old cultures (84.6 ± 1.6%, n = 32 images) (P < 0.01 one-way ANOVA). These data corroborated previous reports demonstrating maturation of the ECM/PNNs during the fourth developmental week *in vitro*^19^,^20^ and showed that maturation of the ECM in neuronal cultures coincides with steady state stabilization of activity levels.

### Maturation of the ECM correlates with formation of network connectivity

**Figure 2:**
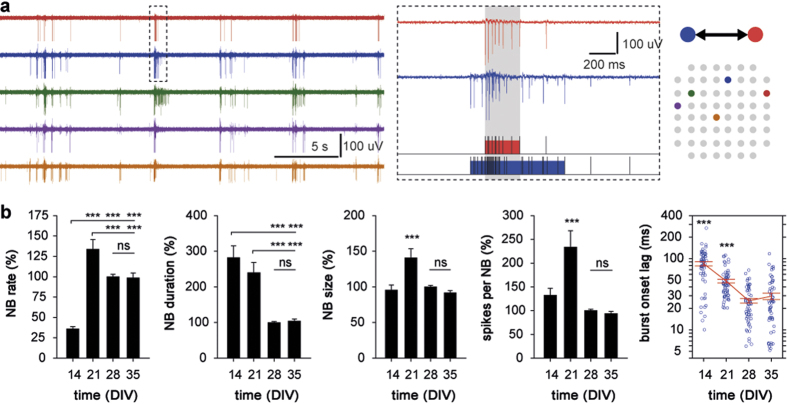
Functional network interaction in hippocampal cultures becomes stereotypic after four weeks *in vitro*. (**A**) Schematic representation of the network burst analysis. Analysis of network interaction was carried out on multi-channel recordings of activity (5 representative channels in the left panel; rat hippocampal culture at DIV21) and was based for detection of non-zero periods of coordinated bursting (gray area in the middle panel) reflecting a functional connection between distinct neuronal clusters. (**B**) The stabilization of all the NB parameters after DIV28 demonstrated that spontaneous formation of the network connectivity requires four weeks *in vitro* and is associated with synchronization of the bursting activity of spatially remote neuronal clusters within 20–30 ms. ***P < 0.001; ns: non-significant (Duncan test). Data are shown as mean ± S.E.M.

Simultaneous multi-site recording of neuronal activity provides the possibility to analyze the dynamic interaction between spatially remote individual neurons or neuronal clusters. Given that bursting becomes a predominant activity pattern in cultured networks upon maturation^25^,^34^,^35^, we evaluated the developmental changes of network (population) bursts, that represent functional interaction between anatomically interconnected neurons. In the present study, network burst (NB) was defined as a non-zero period of correlated bursting derived simultaneously by two or more electrodes (Fig. 2a).

Several spatial and temporal properties of NBs changed significantly during development, including their mean rate, duration and size, as well as the mean number of spikes per NB (all P < 0.001 one-way ANOVA). Again, the particular importance of the third and fourth developmental weeks was evident, as significant changes of NB properties were found only at DIV21 and DIV28 (Fig. 2b). The NBs in three-week old cultures occurred more often, were spread wider apart and comprised of more spikes than at any other developmental stage. In contrast, four week old cultures were characterized by an overall reduction and further stabilization of all NB properties. NBs were less prevalent, of shorter duration and more spatially localized in mature (DIV28-DIV35) cultures compared to three-week old ones. At least partially, changes in rate and duration of NBs could be attributed to corresponding changes of the MBR and the mean burst duration, respectively (Fig. 1b). The similar number of spikes per NB at DIV14 and DIV28, despite a three-fold difference in the mean NB duration, suggested that the synchronization of bursting onset between distant neuronal clusters undergoes a developmental optimization. To examine this, we analyzed the mean burst onset lag between channels sequentially recruited into coordinated bursting (see Methods for details) and found that this parameter was subject to strong developmental regulation (P < 0.001 one-way ANOVA). In contrast to other NB properties, both third and fourth weeks *in vitro* were associated with consistent and steep decreases in mean burst onset lag from 84.8 ± 5.7 ms (DIV14) to 48.3 ± 2.9 ms (DIV21) and further to 25.5 ± 1.8 ms (DIV28), while only marginal change was evident at DIV35 (29.6 ± 3.0 ms) (Fig. 2b).

These results demonstrate that the spontaneous formation of functional connectivity in dissociated hippocampal cultures was complete after four weeks *in vitro*. Since cultured networks are deprived of sensory inputs, the remarkable stereotypy of the network interaction in adult cultures constituted developmental arrest. Furthermore, we found that the synchronization of bursting activity across the network was optimized during development. In mature hippocampal cultures, the recruitment of neuronal clusters into coordinated bursting preferentially occurred within the gamma temporal window (8–30 ms).

### Degradation of the ECM in mature neuronal cultures transiently enhances neuronal activity

**Figure 3:**
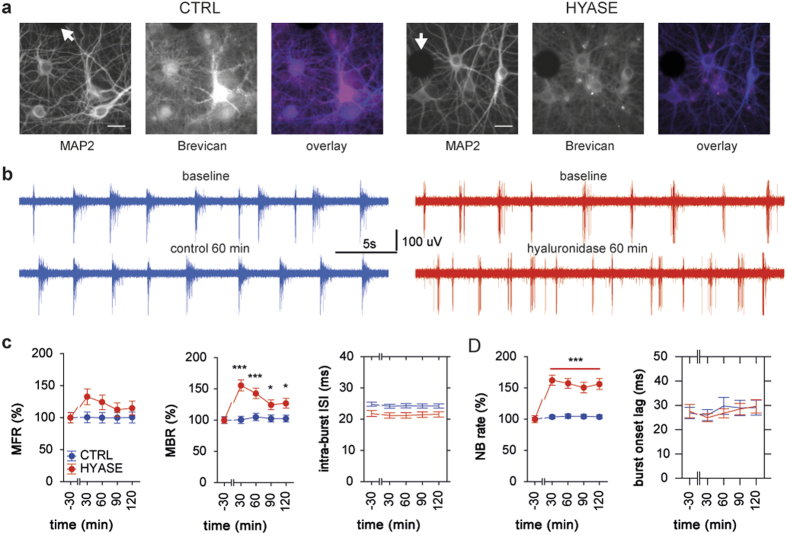
Enzymatic degradation of the ECM by hyaluronidase enhances neuronal activity in mature (DIV28+) hippocampal cultures. (**A**) Representative images of Brevican immunostaining in naïve (CTRL) and hyaluronidase-treated (HYASE; 500 U/mL for 60 min) rat hippocampal cultures grown on MEAs (both DIV35). Black circles correspond to electrode tips (indicated by arrows). Scale bar 20 μm. (**B**) Representative traces of neuronal activity derived from the same channel at corresponding time-points in culture with the intact (blue) and degraded (red) ECM. (**C**) The removal of the ECM elevated the incidence rate of spiking and bursting, while the bursting properties were not affected. (**D**) The application of hyaluronidase induced an increase of the rate, but not of other spatiotemporal properties of the functional network interaction. *P < 0.05, ***P < 0.001; ns: non-significant (Dunnett test). Data are shown as mean ± S.E.M.

Next, we addressed the central question whether removal of the ECM affects the neuronal network activity. The degradation of the ECM in following experiments was carried out exclusively in mature (DIV28+) hippocampal cultures using a single application of hyaluronidase (500 units/mL) that causes quick removal of the ECM (Fig. 3a, Supplementary Fig. S2). Once degraded, ECM recovered within a few days to control pre-treatment levels (Supplementary Fig. S3). Such reconstitution of the ECM could be explained by both temporally restricted enzymatic activity of hyaluronidase (less than 1 hour at 37 °C), confirmed by immunohistochemical and electrophysiological analyses (Supplementary Fig. S4), and culturing in presence of glial cells that play a major role in development of the matrix^36^.

Application of hyaluronidase in mature hippocampal cultures (HYASE group; *n* = 6 MEAs from 5 preparations; DIV28-36) into culture medium led to a pronounced enhancement of neuronal activity (Fig. 3b,c). In immature cultures (*n* = 5 MEAs from 3 preparations; DIV14-17), no significant effect of hyaluronidase on neuronal activity was found (Supplementary Fig. S4). To verify that transient enhancement of neuronal activity was caused by degradation of the ECM, in control cultures (CTRL group; *n* = 6 MEAs from 4 preparations; DIV28-36) we recorded the activity under identical experimental conditions without application of hyaluronidase, and found no significant change over time for any of the analyzed parameters. Analysis showed that the MFR in HYASE group cultures was significantly higher during the post-treatment period, when compared with corresponding values in control cultures (*group* P < 0.01). Bursting rate was strongly affected by the ECM degradation, as MBR in the post-treatment period was higher in HYASE culture groups when compared with either respective baseline values (*treatment* P < 0.001) or corresponding values in control cultures (*group* P < 0.001). No significant effect of the temporal factor or factorial interaction on these parameters was found. The removal of the ECM did not affect the mean burst duration and the mean number of spikes per burst, nor did the mean intra-burst ISI change significantly in any of two groups (Fig. 3c).

In agreement with resemblance of developmental profiles of the MBR (Fig. 1b) and NBR (Fig. 2b), the increase of bursting rate in hyaluronidase-treated cultures was associated with a strong facilitation of network interaction. The rate of NBs was higher upon degradation of the ECM, when compared with respective baseline values (*treatment* P < 0.001), or control cultures with intact ECM (*group* P < 0.001) (Fig. 3d). Notably, this elevation of NBR was not accompanied by changes in bursting synchronization: during baseline period, the mean burst onset lag in cultures of both HYASE (27.6 ± 2.6 ms) and CTRL (26.9 ± 2.5 ms) groups (Fig. 3d) was in the range characteristic for mature cultures (Fig. 2b), and did not significantly change over time. Additionally, treatment with hyaluronidase led to a significant increase of the mean NB duration in cultures of HYASE group (*group* P < 0.05). No significant effect of temporal factor or factorial interaction was found for any of the NB parameters.

Thus, acute removal of the ECM in mature hippocampal cultures led to pronounced enhancement of neuronal activity, suggesting that application of hyaluronidase altered the balance between excitation and inhibition in the network. Intriguingly, the ECM degradation affected only the rate of NBs, leaving other spatiotemporal features of the network interaction unaffected. These outcomes demonstrated that the stereotypic character of the network interaction typical for adult cultures (Fig. 2b) remained as such upon treatment with hyaluronidase, which could be interpreted as the removal of the ECM did not alter the functional connectivity. However, due to deprivation of physiologically relevant inputs, cultured neuronal networks lack any sensory drive for plasticity. Therefore, the ECM degradation *per se* could not provide conclusive evidence for the hypothesized role of the ECM in the retention of network connectivity.

### Degradation of the ECM prevents disinhibition-induced hyperexcitability

To clarify whether enhancement of neuronal activity in hyaluronidase-treated cultures was due to strengthening of excitatory and/or weakening of inhibitory drive, in the next set of experiments we complemented the degradation of the ECM with blockade of fast inhibition by bicuculline (10 μM). Here, disinhibition was employed to dissect the potential impact of the ECM on regulation of excitatory drive in the network, as well as, through induction of hyperexcitability, to provide otherwise missing stimulus for activity-dependent rearrangement of the network connectivity. Once applied, bicuculline was present in the culture medium throughout the recording session (no washout).

**Figure 4:**
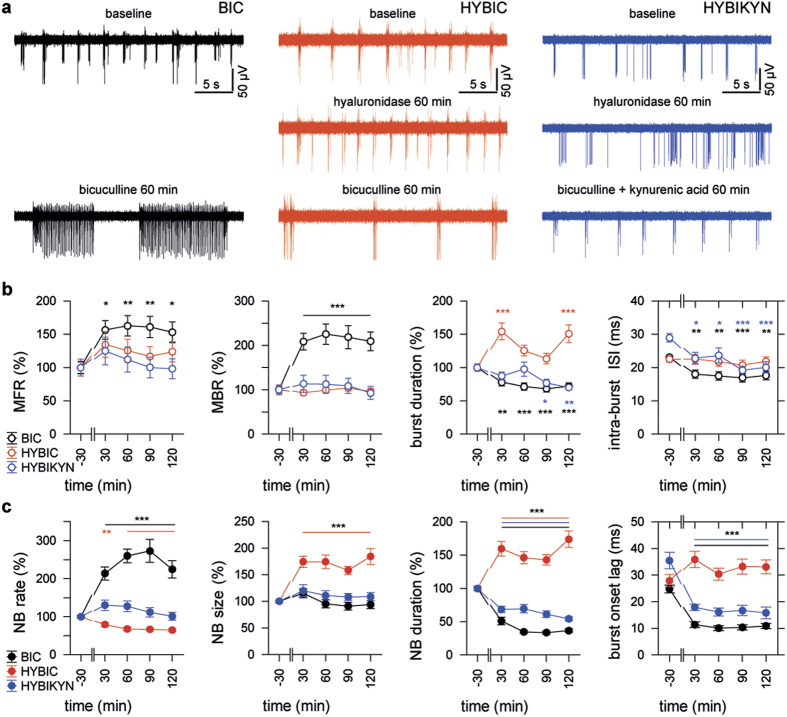
The lack of disinhibition-triggered hyperexcitability in cultures with degraded ECM is associated with desensitization of glutamate receptors. (**A**) Representative traces of activity recorded prior to and after application of bicuculline in cultures with the intact ECM (BIC, black), as well as with degraded ECM either alone (HYBIC, red), or in combination with kynurenic acid (HIBIKYN, blue). (**B**) The effect of disinhibition on the spiking and bursting activity was virtually abolished by the degradation of the ECM 1 h prior to application of bicuculline, but was partially restored when induced in presence of kynurenic acid. (**C**) The disinhibition in cultures of HYBIC group was characterized by the pattern of network interaction remarkably different from that in cultures of BIC and HYBIKYN groups, and was not associated with hypersynchronization of bursting activity across the network. *P < 0.05, **P < 0.01, ***P < 0.001; ns: non-significant (Dunnett test, colours of asterisks correspond to groups). Data are shown as mean ± S.E.M.

As expected, disinhibition in naïve hippocampal cultures (BIC group; *n* = 5 MEAs from 4 preparations; DIV28-38) led to dramatic enhancement of both MFR and MBR (*treatment* P < 0.01 and P < 0.001, respectively) (Fig. 4a,b). Bicuculline induced a characteristic epileptiform pattern with short bursts occurring in trains lasting up to tens of seconds and a significant decrease in the mean burst duration (*treatment* P < 0.001) (Fig. 4b). Furthermore, blockade of fast inhibition in cultures with the intact ECM led to marked reduction of the mean intra-burst ISI from 23.1 ± 0.7 ms during baseline to 17.5 ± 0.6 ms in post-treatment period (*treatment* P < 0.001).

Disinhibition in cultures pre-treated with hyaluronidase for 60 min (HYBIC; *n* = 6 MEAs from 5 preparations; DIV28-42) induced a pattern of activity that was remarkably different from that seen in cultures of either HYASE, or BIC groups. In striking contrast to cultures with intact ECM, application of bicuculline in cultures with degraded ECM failed to increase spiking and bursting rates, but increased the mean burst duration (*treatment* P < 0.001). In contrast to the BIC group, disinhibition in cultures with degraded ECM did not affect frequency of intra-burst firing (Fig. 4b).

Previously, the ECM was proposed to act as a microenvironment that can modulate ambient glutamate concentration in mature synaptic contacts^10^. We assumed that apparent failure of bicuculline to induce epileptiform activity in cultures with degraded ECM was associated with spillover of glutamate, which leads to activation and following desensitization of glutamate receptors in peri- and extrasynaptic locations^37^,^38^. It should be noted that, as opposed to standard low density culture preparations on coverslips, high density neuronal cultures form multilayered cellular networks (Supplementary Video S6). In additional group of cultures, we applied bicuculline together with a non-saturating concentration of kynurenic acid, a mild competitive antagonist of ionotropic glutamate receptors^39^. The combined application of bicuculline and kynurenic acid (100 μM) in cultures with the degraded ECM (HYBIKYN; n = 4 MEAs from 4 preparations; DIV29-35) induced a regular bursting pattern (Fig. 4a), which was not associated with significant changes of firing or bursting rates, as compared to baseline values. However, the mean intra-burst ISI significantly decreased from 29.0 ± 1.3 ms during baseline to 21.5 ± 0.9 ms in post-treatment period (*treatment* P < 0.001), which coincided with the marked shortening of the mean burst duration (*treatment* P < 0.01). The antagonism of glutamate receptors in cultures with degraded ECM but without bicuculline induced marked reduction of firing rate, but did not affect bursting parameters (Supplementary Fig. S5).

The effects of disinhibition strongly varied between groups for MFR, MBR, mean burst duration and mean intra-burst ISI (*group* all P < 0.001). No significant effects of temporal factor and factorial interaction were found. Cross-group comparison showed that the mean MFR, MBR and intra-burst ISI in cultures of BIC group were markedly different in post-treatment period, when compared with respective values in cultures of either HYBIC (P < 0.05, P < 0.001 and P < 0.001, respectively; Duncan test), or HYBIKYN (all P < 0.001 Duncan test) groups. No differences were evident for these parameters between cultures of HYBIC and HYBIKYN groups. However, antagonism of glutamate receptors by kynurenic acid led to shortening of the mean burst duration in cultures of HYBIKYN group in comparison with respective values in cultures of HYBIC group (P < 0.001 Duncan test).

Further, we found that the ECM integrity significantly influenced the properties of neuronal network interaction in presence of bicuculline. Disinhibition in naïve cultures increased the incidence rate of NBs whereas in disinhibited cultures pre-treated with hyaluronidase NBs were less frequent, but lasted longer and propagated into a larger number of network locations (*treatment* all P < 0.001). Similar to the effect of bicuculline in naïve cultures, disinhibition in the presence of kynurenic acid in HYBIKYN group cultures led to a significant decrease of the mean NB duration (*treatment* P < 0.001) without affecting the mean NB size (Fig. 4c). No significant effect of treatment on the NBR in cultures of HYBIKYN group was found, contrasting to significant reduction of all NB properties seen in cultures treated with hyaluronidase and kynurenic acid but without bicuculline (Supplementary Fig. S5).

The results of two-way ANOVA revealed strong impact of the ECM degradation on the effect of disinhibition for all analyzed NB properties (*group* all P < 0.001). Between-group differences were significant for all parameters, except the difference in the NB size between BIC and HYBIKYN groups (Duncan test). These data demonstrated that kynurenic acid, when applied to cultures with degraded ECM, to a large degree restored the effect of bicuculline on the functional network interaction. Importantly, both the ECM integrity and the antagonism of glutamate receptors strongly influenced the synchronization of bursting activity (Fig. 4c). In cultures of BIC and HYBIKYN groups, the mean burst onset lag (24.8 ± 1.5 ms and 35.5 ± 3.1 ms during baseline, respectively) was significantly reduced by application of bicuculline into culture medium (10.7 ± 0.5 ms and 16.7 ± 0.9 ms, respectively) (*treatment* both P < 0.001). In contrast, the synchronization of bursting activity in cultures of HYBIC group (27.9 ± 2.3 ms during baseline) was not affected by application of bicuculline (33.1 ± 1.4 ms in post-treatment period). Furthermore, enzymatic removal of the ECM prevented the bicuculline-driven hypersynchronization of bursting activity across the network (*group* P < 0.001), resulting in significant difference between BIC and HYBIC groups (P < 0.001 Duncan test). The mean burst onset lag in cultures of HYBIKYN group was markedly different in comparison with those of either BIC or HYBIC groups (both P < 0.001 Duncan test). Thus, antagonism of glutamate receptors led to partial rescue of bicuculline-triggered hypersynchronization of bursting in disinhibited cultures with degraded ECM.

Taken together, these findings showed that the blockade of fast inhibition in cultures with degraded ECM evoked distinct patterns of network activity that could not be attributed to the effect of either hyaluronidase, or bicuculline alone. Given that the blockade of GABAA receptors by bicuculline is independent of ECM integrity, the dramatic difference in network activity between disinhibited cultures with intact or degraded ECM demonstrates that the ECM in mature neuronal cultures modulates excitatory neurotransmission.

## Discussion

As a model of neuronal networks, dissociated cultures are inherently artificial due to their chronic isolation from relevant external inputs. This may be considered a significant disadvantage of this preparation for some applications, however in the context of our study such sensory deprivation was absolutely essential and allowed us to gain insight into truly intrinsic mechanisms of formation and maintenance of connectivity. Cultured neuronal networks are not influenced by the ever-present sensory inflow which drives network activity in intact brain from the early postnatal period^8^ and can easily mask the network effects of ECM degradation. Additionally, the accessibility of cultures for imaging allowed us to assess both functional and structural consequences of controlled ECM degradation in the same experimental system. Taken together, these aspects rendered the dissociated neuronal cultures grown on MEAs as the most adequate system for evaluation of the role of extracellular matrix in the maintenance of network connectivity.

The analysis of the developmental profile of spontaneous network activity revealed that the third week *in vitro* was associated with high network excitability, whereas during the fourth week there was a global reduction and steady state stabilization of neuronal activity levels. A near-universal developmental trend, with initial exuberance of excitatory synaptic contacts followed by maturation of GABAergic synapses and elimination of excessive glutamatergic synapses^1^,^32^, strongly suggests that synaptic connectivity to a certain extent is a product of neuronal activity in a given network during development^40^. Indeed, manipulation of neuronal activity in developing cultured networks strongly affects their maturation^3^, with a particularly strong impact on inhibitory connections^41^. Suppression of neuronal activity in developing cultures delays^29^, while enhancement of neuronal activity, induced by disinhibition^42^ or application of BDNF^43^ accelerates the maturation of cultured neuronal networks. Thus, high network excitability characteristic for immature networks seems to represent a natural mechanism promoting the development of connectivity^1^ by providing sufficient depolarization of inhibitory interneurons needed for maturation of GABAergic connectivity.

In line with this suggestion and consistent with earlier reports^34^,^35^, we found two transitional steps in the developmental profile of network bursts. Large increases in the rate, area and duration of NBs during the third week *in vitro* were followed by a significant reduction of all three measures by the end of fourth week, coinciding with the appearance of a mature pattern of the ECM. Although our results provided no evidence of causality, treatment of immature (DIV17) hippocampal cultures with hyaluronidase has been reported to induce long-term hyperexcitability characterized by the presence of generalized long-lasting epileptiform discharges^44^. This indicates that the activity-dependent formation of ECM/PNNs^19^ might aid in the maturation of the inhibitory system, and interference with the ECM formation early in development affects the establishment of balance between excitation and inhibition. Furthermore, developmental (P6-P25) increase of excitability and responsiveness of Parvalbumin-positive interneurons that are surrounded by particularly dense PNNs^18^,^21^,^22^ were reported to increase during development (P6-P25)^45^, suggesting that contribution of the ECM to development of inhibitory system might be exerted by regulation of excitatory inputs onto GABAergic interneurons.

In this study, we hypothesized that the restriction of developmental plasticity by hyaluronan-based ECM^46^ is related to its role in retention of existing network connectivity. The analysis of disinhibition-triggered effects in cultures with degraded ECM supported this hypothesis. We found that removal of the ECM prior to application of bicuculline prevented hyperexcitability and virtually abolished the manifestation of characteristic epileptiform discharges that were abundantly present in disinhibited cultures with intact ECM. Central synapses were reported to operate on a principle of resource optimization aimed to maximize the postsynaptic response per released molecule of glutamate^47^. Our results suggest that regulation of ambient glutamate concentration by the ECM might be an important contributing factor for such optimization, and ECM degradation favours glutamate spillover. Furthermore, the ECM has been shown to act as a physical barrier restricting the lateral mobility and exchange rate of naïve and desensitized AMPA receptors between synaptic and extrasynaptic locations^48^, thus competitive antagonism of glutamate receptors would play a protective role against their desensitization. Indeed, we found that kynurenic acid counterbalanced the effect of ECM removal and partially restored the effects of disinhibition in cultures with degraded ECM. These data revealed an additional functional implication of synaptic confinement of glutamate by the ECM, namely the protection of extrasynaptic pool of receptors from exposure to glutamate that would lead to their activation and further desensitization^37^,^38^.

Remarkably, disinhibition in cultures with degraded ECM induced distinct changes of the spatiotemporal properties of neuronal activity reflecting the global shift towards longer and more global patterns of the network activity. We found that mature disinhibited cultures with degraded ECM (Fig. 4c) shared all the features of NBs in naïve developing cultures (Fig. 2c), indicating similar prevalence of excitation over inhibition and suggesting substantial backward shift in developmental state upon ECM removal. Such a re-emergence of the juvenile pattern of network activity might be beneficial for activity-dependent remodelling of connectivity, so that longer periods of coordinated bursting within a broader synchronization window could recruit a larger number of neuronal clusters and thereby promote the exploration of novel network architecture. Considering disinhibition as a challenge for neuronal networks in taming excessive excitation and readjusting the balance between excitation and inhibition, these results demonstrate that experimental degradation of the matrix facilitated disinhibition-driven changes in functional network interaction. Notably, single cell recordings did not reveal significant effect of ECM degradation on the resting membrane potential, spiking threshold and action potential shape, as well as miniature EPSCs in hyaluronidase-treated cultures^48^,^49^ and slices^50^. We also found the effect of the hyaluronidase treatment on MFR was rather minor, whereas it was particularly strong on the network interaction that requires active participation of interneurons.

Although outnumbered, GABAergic interneurons govern the activity of pyramidal neurons via various mechanisms^51^. Interneurons regulate the temporal precision of principal cell firing in the millisecond-range^52^,^53^ and mediate long-range connectivity^54^. The developmental maturation of inhibitory connectivity is therefore likely associated with improved temporal coordination of activity and facilitation of network interaction within spatially distributed neuronal ensembles. Indeed, we found that cultures exhibited a dramatic reduction of the mean burst onset lag during the third and fourth weeks from ~85 ms at DIV14 to ~25–30 ms at DIV28. This provides much needed context for the previously reported developmental increase of gamma frequency in freely moving rats^55^ and alterations of theta and gamma oscillations in Tenascin-R knockout mice^56^. Results from our neuronal cultures support the relationship between ECM-dependent regulation of excitability of interneurons and improved temporal coordination of the network activity in hippocampal cultures upon maturation. Through optimization of excitatory inputs onto basket cells that play a crucial role in genesis and maintenance of hippocampal gamma oscillations^57^, the ECM might promote rhythmic inhibition of principal neurons and the dynamic activity-dependent modulation of their firing within the gamma window^58^.

The coordination of network interaction according to ongoing gamma oscillatory cycle, e.g. arising through “stochastic synchrony” in the spike output of single neurons that receive noisy inputs^59^, might represent a default interaction mode within functional ensembles of hippocampal neurons. Such an intrinsic mechanism of spontaneous formation and maintenance of network connectivity via gamma interaction shapes hippocampal cultures as “standby” networks: despite the lack of relevant sensory inputs, they nevertheless develop and optimize their connectivity in a self-organized manner into a stable and relatively idle state. The results of our study demonstrate that the appearance of hyaluronan-based ECM plays an important role in the retention of such functional connectivity established during development.

## Methods

### Ethics Statement

All experimental procedures were carried out in accordance with the EU Council Directive 86/609/EEC and were approved and authorised by the local Committee for Ethics and Animal Research (Landesverwaltungsamt Halle, Germany).

### Dissociated neuronal cultures

Neuronal cultures were prepared from Wistar rat embryos (E18) as described previously^60^. For multichannel recordings, suspension of dissociated hippocampal cells (500,000–750,000 cells/mL) was plated on Poly-D-lysine-coated 60-channel (inter-electrode distance 200 μm) MEAs (MultiChannel Systems, Reutlingen, Germany). After plating, all cultures were incubated in serum-free Neurobasal medium at 37 °C in a humidified atmosphere (95% air and 5% CO_2_), covered by semi-permeable membranes (ALA-MEM, MultiChannel Systems) to avoid evaporation of the medium. Culture medium was partially replaced on a weekly basis by astrocyte-conditioned medium.

### Compounds and treatments

Application of compounds to cultures was carried out exclusively after DIV28. Prior to any treatment, the baseline recording of spontaneous neuronal activity was carried out in all cultures for at least one hour. Stock solutions of hyaluronidase (Sigma-Aldrich; H4272, type IV-S from bovine testes), bicuculline methiodide (Sigma-Aldrich; 14343) and kynurenic acid (Sigma-Aldrich; K3375) were applied in volumes between 2 and 10 μL, so that the final concentration of compounds in the MEA well was 500 U/mL, 10 μM and 100 μM, respectively. Once applied, all compounds remained in the medium (no washout). No perfusion system was used whatsoever.

### Immunocitochemistry

The immunostaining of Aggrecan and Brevican was performed in rat hippocampal cultures grown on MEAs using rabbit anti-Aggrecan polyclonal antibody (sera 1:500; Merck Millipore) and custom made guinea pig anti-Brevican antibody (sera 1:1000), respectively. After fixation in phosphate-buffered saline (PBS, pH 7.4) containing 4% paraformaldehyde and 4% sucrose for 15 min, samples were blocked for 60 min (10% FCS, 0.1% glycine and 0.1% Triton in PBS). Next, cultures were incubated with primary mouse anti-MAP monoclonal antibody (sera 1:1000; Synaptic Systems) for 60 min at room temperature, washed with PBS (3 × 5 min), and incubated with secondary antibodies (goat anti-guinea pig Alexa647, goat anti-mouse Cy5 and goat anti-rabbit Cy3; Invitrogen) for 60 min. After final washing with PBS (3 × 5 min), cultures were mounted in Mowiol (Hoechst). Image acquisition was carried out using Olympus IX71 microscope with the 60× UPlanSApo oil objective and the Andor Luca R camera (Andor, Northern Ireland) and MetaMorph software (Molecular Devices, USA). Images were acquired as Z-stacks of 10 or 21 planes (spacing between planes 0.5 μm) in cultures grown on coverslips and MEAs, respectively, and subsequently used for calculation of the average fluorescence intensity. Further, images with MAP2 signal were binarized and used as a mask for quantification of the fluorescence intensity of Brevican or Aggrecan. Analysis of immunofluorescence was performed using ImageJ software (NIH, USA).

### Recording and analysis of neuronal network activity

The neuronal activity was sampled at 10 kHz using MC_Rack software and MEA1060INV-BC system (MultiChannel Systems, Reutlingen, Germany) placed inside of incubator (Unitherm 3503-2; Uniequip, Germany) that provided identical conditions (temperature, humidity and gas composition) to those in the culturing incubator. To avoid movement-induced artefacts, any activity recorded within first 20 min after physical translocation of MEAs from incubator to incubator was discarded from further analyses. The off-line analysis was carried out on 600-sec long sessions for each culture at each time-point. The detection of spikes ( ± 6 SDs) in high-pass (300 Hz) filtered records was followed by identification of bursts (≥5 spikes with inter-spike interval (ISI) ≤ 100 ms). Taking into account relatively large size of electrodes (30 μm), no spike sorting algorithms were employed, and detected spikes were analyzed as point processes (i.e., neither shape, nor amplitude of spikes was taken into consideration). Channels with the mean firing rate (MFR) < 0.1 Hz were considered as non-spiking in given session and discarded from further analyses. Channels with the mean bursting rate (MBR) < 1 burst/min were considered as non-bursting (but not necessarily non-spiking) and were not used for calculation of bursting parameters. The MFR and MBR, as well as the mean burst duration and the mean number of spikes per burst, were calculated separately for each active channel (electrode) in each individual culture. Coefficient of variation (CV) of was computed as CV = SD/mean.

Network burst (NB) was defined as a non-zero period of correlated (synchronous) bursting in two or more channels. For each NB, participating channels were ranked by burst onset time according to temporal order of their recruitment into coordinated bursting, thus forming vector (*1*, …, *n*), where *n* denotes the rank of the last recruited channel (i.e., the size of given NB; n ≥ 2). Multiple participation of the same channel in given NB was allowed. The mean burst onset lag characterizing the synchronicity of bursting onset between remote network locations was calculated for each NB as 
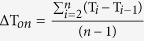
, where T_*i*_ denotes the burst onset time in channel with rank *i* within given NB. The calculation of NB properties, including the NB rate (NBR), the NB duration, NB size, as well as the number of spikes emitted by participating channels within NB episodes, was carried out per minute for 600-s long sessions at each time-point. Signal processing and all analyses of neuronal activity were carried out using Spike2 software (Cambridge Electronic Design, Cambridge, UK).

### Statistics

For better comparability of experimental outcomes across the study, the developmental data for each individual culture were normalized to respective mean value obtained at DIV28 (taken as 100%). In the rest of experiments, data were normalized to the mean value obtained from baseline (control) session recorded 30 min prior to any treatment. The effects of *development* (4 levels corresponding to DIV14-35) and *treatment* (5 levels corresponding to baseline period and 4 post-treatment time-points) factors were analyzed using protected one-way ANOVA followed by Duncan’s and Dunnett *post hoc* tests, respectively. The effects of *group* and *time* factors, as well as of their interaction, were evaluated in post-treatment data using protected two-way ANOVA followed by Duncan’s *post hoc* test. Treatment of data and statistical analysis were performed using STATISTICA data analysis system (Statsoft, Inc., Tulsa, USA). Factorial effects and differences were considered as significant at P < 0.05. Data are presented as mean ± S.E.M., unless mentioned otherwise.

## Additional Information

**How to cite this article**: Bikbaev, A. *et al.* Brain extracellular matrix retains connectivity in neuronal networks. *Sci. Rep.* **5**, 14527; doi: 10.1038/srep14527 (2015).

## Supplementary Material

Supplementary Information

Supplementary Video S6
