# Supplementary material for: Brain extracellular matrix retains connectivity in neuronal networks
Source: Sci Rep. 2015 Sep 29;5:14527. doi: 10.1038/srep14527 (PMC4586818; doi:10.1038/srep14527)
Supplement: Supplementary Information [file srep14527-s1.doc]

**Brain extracellular matrix retains connectivity in neuronal networks**

Arthur Bikbaev^1^, Renato Frischknecht^2^ & Martin Heine^1^

^1^RG Molecular Physiology, Leibniz Institute for Neurobiology; Brenneckestr. 6, Magdeburg 39118 Germany

^2^RG Brain Extracellular Matrix, Leibniz Institute for Neurobiology; Brenneckestr. 6, Magdeburg 39118 Germany

*Corresponding authors:*

Dr. Arthur Bikbaev, e-mail: [abikbaev@lin-magdeburg.de](mailto:abikbaev@lin-magdeburg.de)

RG Molecular Physiology, Leibniz Institute for Neurobiology; Brenneckestr. 6, Magdeburg 39118 Germany; +49-391-6263-93181

Dr. Martin Heine, e-mail: [mheine@lin-magdeburg.de](mailto:mheine@lin-magdeburg.de)

RG Molecular Physiology, Leibniz Institute for Neurobiology; Brenneckestr. 6, Magdeburg 39118 Germany; +49-391-6263-93361

**Supplementary Information**

**Supplementary Figures**

**Supplementary Figure S1**

**
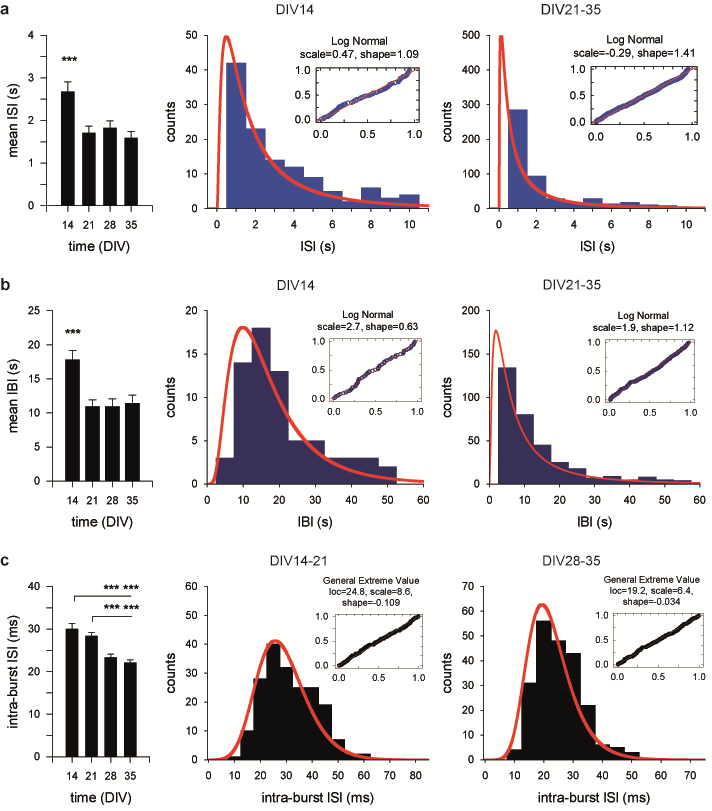
**

**Supplementary Figure S2**

**
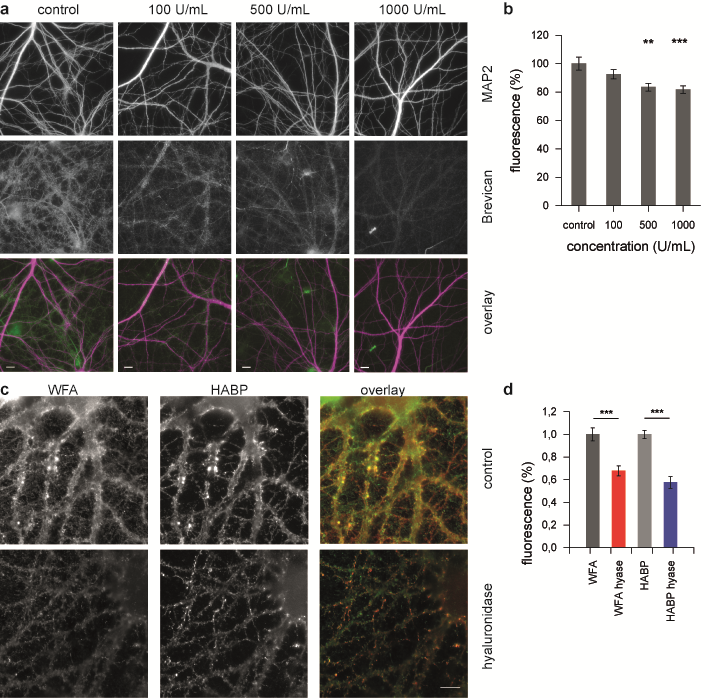
**

**Supplementary Figure S3**

**
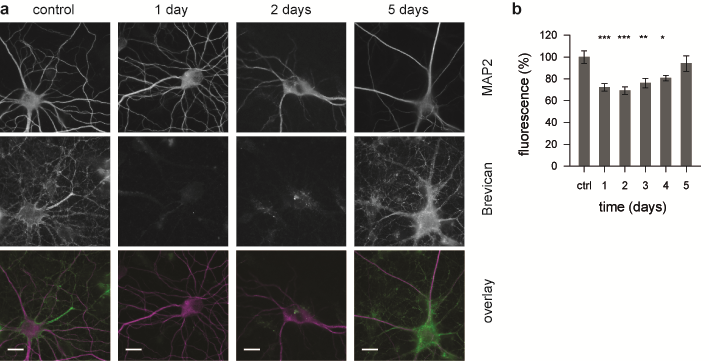
**

**Supplementary Figure S4**

**
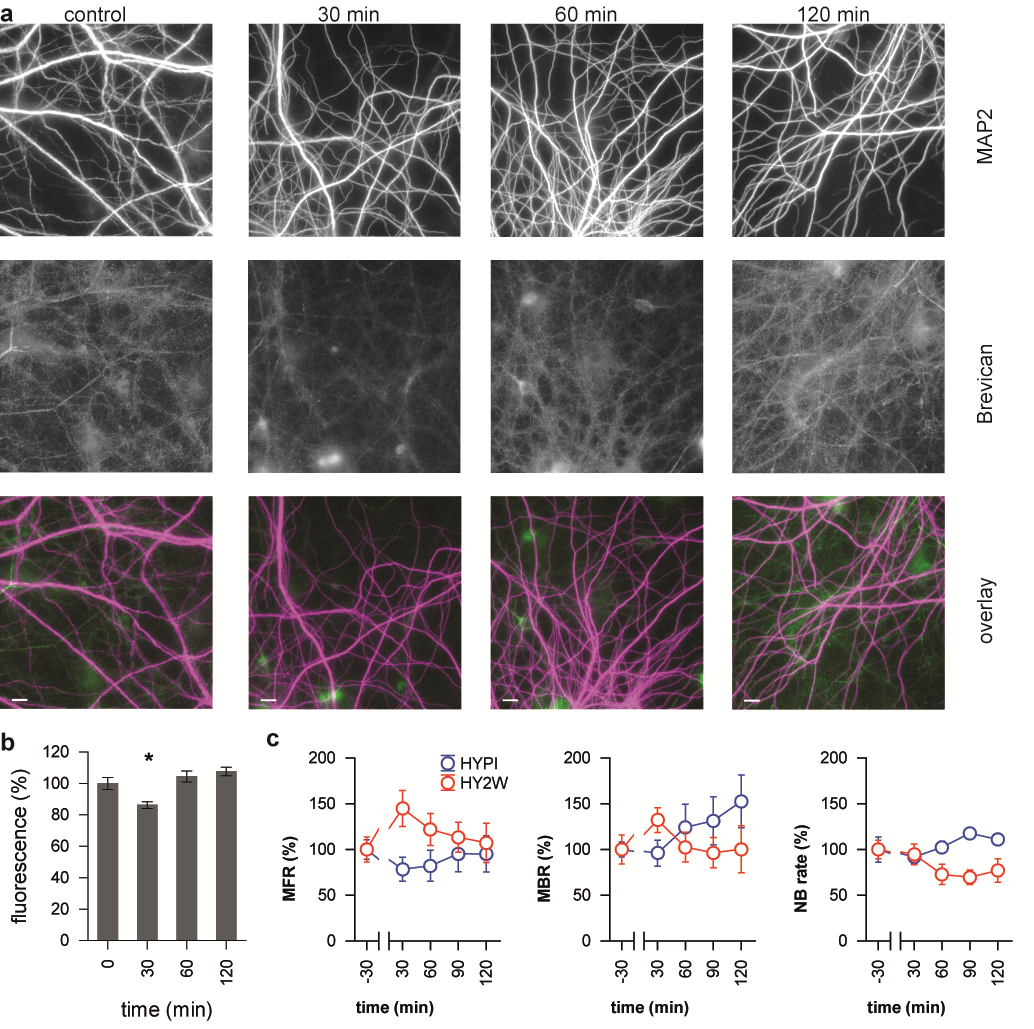
**

**Supplementary Figure S5**


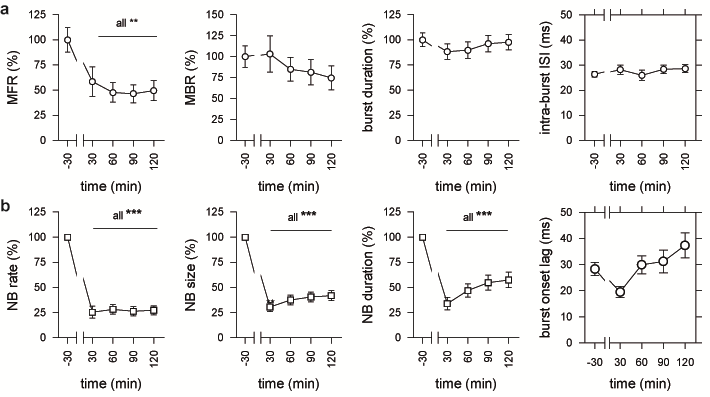


**Supplementary Video S6**

**Supplementary Figure Legends**

**Supplementary Figure S1.** **Characterization of variability in spontaneous spike trains recorded in dissociated hippocampal cultures during development. A.** The effect of developmental factor on the mean ISI (P < 0.001 one-way ANOVA) was associated with its marked decrease during the third week *in vitro*. Log-normal distribution was characteristic for the mean ISI (both DIV14 and pooled DIV21-35: P > 0.05 Kolmogorov-Smirnov (K-S) test, P > 0.05 χ^2^ test). **B.** The mean IBI was subject to the developmental regulation (P < 0.001 one-way ANOVA), with the log-normal distribution being characteristic for IBIs at all developmental stages (both DIV14 and DIV21-35: P > 0.05 K-S test, P > 0.05 χ^2^ test). **C.** The mean intra-burst ISI changed markedly during development (P < 0.001 one-way ANOVA). The highest probability of mean intra-burst ISI values was in the range of 20-30 ms (generalized extreme value distribution; P > 0.05 K-S test, P > 0.05 χ^2^ test for both pooled datasets DIV14-21 and DIV28-35). *** P < 0.001 (Duncan test). Data are shown as mean ± S.E.M.

**Supplementary Figure S2. Treatment with hyaluronidase results in dose-dependent degradation of the hyaluronan-based ECM in dissociated neuronal cultures. A.** The immunoreactivity of Brevican in mature rat cortical cultures treated with hyaluronidase at different concentrations, as well as in control non-treated cultures. **B.** Hyaluronidase significantly decreased the mean fluorescence of Brevican (P < 0.001 one-way ANOVA) when applied at concentrations 500 U/mL (83.4 ± 2.7%, n = 24 images) and 1000 U/ml (81.8 ± 2.7%, n = 24 images), but not 100 U/mL (92.6 ± 3.2%, n = 24 images), as compared with untreated controls (100.0 ± 4.6%, n = 26 images). ******* P < 0.001, ** P < 0.01 (Dunnett test). **C.** Examples of immunostaining of the chondroitin sulphate (*Wisteria* *floribunda* agglutinin, WFA) and hyaluronic acid (hyaluronic acid binding protein, HABP) in control and hyaluronidase-treated rat cortical cultures (DIV28). **C.** Quantification of hyaluronidase effect on the WFA or HABP staining (WFA 0.68 ± 0.044, n = 12; HABP 0.58 ± 0.052, n = 9) in comparison with control cultures (WFA 1.00 ± 0.057; HABP 1.00 ± 0.036, both n = 12). *** P < 0.001 (t-test). Scale bar 10 µm. Data are shown as mean ± S.E.M.

**Supplementary Figure S3. Enzymatic degradation of the hyaluronan-based ECM in dissociated neuronal cultures is followed by its spontaneous recovery. A.** Immunoreactivity of Brevican in mature rat cortical cultures treated with hyaluronidase for 1-5 days (n = 7-10 images) prior to staining, as well as in control non-treated cultures (n = 14 images). Scale bar 10 µm. **B.** Application of hyaluronidase (500 U/mL) caused a significant reduction of the mean fluorescence of Brevican (P < 0.001 one-way ANOVA), however it recovered to control levels within 5 days after treatment. *** P < 0.001, ** P < 0.01, * P < 0.05 (Dunnett test). Data are shown as mean ± S.E.M.

**Supplementary Figure S4. Enzymatic activity of hyaluronidase is restricted to 60 min at 37°C. A.** Representative images of Brevican immunostaining in mature rat cortical cultures treated with hyaluronidase, which was pre-incubated at 37°C for various time intervals prior to application (n = 30-32 images), as well as in control non-treated cultures (n = 32 images). Scale bar 10 µm. **B.** Application of hyaluronidase (500 U/mL) pre-incubated at 37°C for 60 min or longer was not associated with significant decrease of the mean fluorescence of Brevican, as compared to controls. * P < 0.05 (Dunnett test). **C.** Neuronal activity and network interaction were not affected by the application of either inactivated hyaluronidase (60 min at 37°C prior to application) in mature hippocampal cultures (n = 5, DIV29-32; MFR P = 0.88, MBR P = 0.47, NB rate P = 0.22; one-way ANOVA), or application of active hyaluronidase in immature cultures (n = 5, DIV14-17; MFR P = 0.40, MBR P = 0.48, NB rate P = 0.16; one-way ANOVA). Data are shown as mean ± S.E.M.

**Supplementary Figure S5. Application of kynurenic acid in neuronal cultures with degraded ECM results in suppression of neuronal network activity. A.** Antagonism of ionotropic glutamate receptors by kynurenic acid in mature rat hippocampal cultures (n = 5 MEAs from 3 preparations; DIV28-31) pre-treated for 60 min with hyaluronidase resulted in suppression of the mean firing rate (P < 0.001 one-way ANOVA) without affecting bursting properties. **B.** Application of kynurenic acid in cultures with degraded ECM affected several properties of functional network interaction, including the mean rate of NBs (P < 0.00001), their size (P < 0.00001) and duration (P < 0.00001), as well as the mean burst onset lag (P < 0.05 one-way ANOVA). *** P < 0.001, ** P < 0.01 (Dunnett test). Data are shown as mean ± S.E.M.

**Supplementary Video S6. High-density neuronal cultures grown on MEAs form multilayered three-dimensional networks** (immunolabelling of MAP2 in rat hippocampal culture at DIV35). Video sequence was converted at 2 frames/s from Z-stack with 21 planes (spacing 0.5 µm) acquired at magnification 60x. Arrow indicates a recording electrode. Scale bar 20 µm.
